# Interpretable predictive model for deterioration of kidney function in patients with stage 4 cardiovascular-kidney-metabolic syndrome

**DOI:** 10.1186/s12882-026-05001-0

**Published:** 2026-04-20

**Authors:** Fangyu Wang, Zhi Shang, Yueming Gao, Zhenling Deng, Wen Tang, Yue Wang

**Affiliations:** 1 Department of Nephrology, Peking University Third Hospital, 49 Huayuan North Road, Haidian District, Beijing, 100191 China; 2 Department of Cardiology and Institute of Vascular Medicine, Peking University Third Hospital, Beijing, 100191 China

**Keywords:** Cardiovascular-kidney-metabolic syndrome, Kidney function, Random survival forest, Prediction model

## Abstract

**Background:**

Cardiovascular-kidney-metabolic (CKM) syndrome is a progressive disease that can affect multiple vital organs. The specific factors contributing to the deterioration of kidney function in stage 4 CKM patients remain unclear, and no relevant clinical prediction model has been established.

**Methods:**

A retrospective analysis was conducted on eleven years of inpatient data. Stage 4 CKM Patients who fulfilled the diagnostic criteria and did not meet the exclusion criteria were enrolled. The outcome was kidney function progression, defined as a sustained decline in estimated glomerular filtration rate of ≥ 40% from baseline or initiation of kidney replacement therapy. Based on clinical indicators, predictive models were constructed using Cox regression, LASSO-Cox regression and random survival forest algorithms. Calibration curves, receiver operating characteristic curves, and decision curve analysis were employed to validate the models. The SHapley Additive exPlanations method was used to interpret the final model. Based on the model, a web-based risk calculator was constructed for clinical practice.

**Results:**

A total of 23,014 subjects with stage 4 CKM were included and randomly divided into two cohorts at a ratio of 2:1 to the development cohort and the validation cohort. During follow-up, 1,772 outcomes (11.6%) occurred in the training cohort and 942 (12.3%) in the validation cohort. Key predictors of kidney function deterioration in stage 4 CKM patients included N-terminal pro-B-type natriuretic peptide, serum albumin, left ventricular ejection fraction, hemoglobin, age, blood urea nitrogen, and urinary protein level. Among the three models, the random survival forest model demonstrated the best discrimination and calibration in the validation set (area under the curve = 0.930, 95% confidence interval: 0.925–0.934 in 12 months).

**Conclusions:**

The random survival forest model was highly accurate in identifying the special patients with an elevated risk of kidney function deterioration in stage 4 CKM syndrome patients. Our model can be utilized for prospective monitoring and support personalized management strategies for this high-risk population.

**Clinical trial number:**

Not applicable.

**Supplementary Information:**

The online version contains supplementary material available at 10.1186/s12882-026-05001-0.

## Introduction 

Cardiovascular-kidney-metabolic (CKM) syndrome is defined as a group of systemic diseases caused by the pathological and physiological interactions between metabolic diseases, chronic kidney disease (CKD), and cardiovascular disease (CVD), leading to multi-organ dysfunction and a high rate of adverse cardiovascular outcomes [1–3]. CKM syndrome affects many people, imposing a significant burden on society and drawing high attention from multidisciplinary experts [4].

In the CKM syndrome, stage 4 is considered as a special stage. A recent survey indicates that about 9.2% of adults met criteria for stage 4 [5]. Patients at this stage already have clinical CVD and may also have CKD and/or metabolic abnormalities [1]. The presence of kidney failure has a significant impact on the treatment and management of the disease at this stage [1, 2]. In addition, patients in this stage face a high risk of kidney function deterioration, potentially progressing rapidly to kidney failure that requires kidney replacement therapy [6]. Therefore, a comprehensive assessment of kidney function and the factors influencing its future trajectory is essential in disease management. Consequently, developing prediction models based on clinical indicators in stage 4 CKM may help healthcare providers facilitate risk stratification and provide personalized interventions accordingly [7].

Although prediction models for kidney function deterioration have been developed in some studies, such as the CKD-PC equation and KidneyInterX [8–11]. Predictive models specifically for stage 4 CKM patients, particularly those based on clinical indicators and derived from large sample sizes, are uncommon. On the other hand, interpretability is equally crucial in clinical prediction, as it facilitates clinicians’ understanding of results and guides clinical practice [12]. However, interpretable predictive models in stage 4 CKM patients are lacking. Therefore, we aimed to develop and validate a model to predict subsequent kidney function deterioration in patients with stage 4 CKM syndrome that could be easily implemented in clinical practice, using the variables routinely measured in patients with CKM.

## Methods

### Study population and design

This study retrospectively collected clinical data from 62,374 patients hospitalized at Peking University Third Hospital from January 1, 2013, to December 31, 2023. The inclusion criteria included: (1) age ≥ 18 years; (2) meeting the diagnostic criteria for CKM syndrome stage 4. The diagnostic criteria for CKM syndrome stage 1–4 are as follows: stage 1: individuals with excess and/or dysfunctional adiposity, including body mass index (BMI) ≥ 23 kg/m^2^, waist circumference (WC), ≥ 80 cm in women or ≥ 90 cm in men, fasting blood glucose (FBG) ≥ 100–124 mg/dL, or glycosylated hemoglobin (HbA1c) between 5.7% and 6.4%, without the presence of other metabolic risk factors or CKD; stage 2: individuals with metabolic risk factors, including triglyceride (TG) ≥ 135 mg/dL, hypertension, metabolic syndrome (MetS), or diabetes mellitus (DM) and/or CKD; Stage 3: subclinical atherosclerotic CVD or subclinical heart failure (HF) among individuals with excess/dysfunctional adiposity, other metabolic risk factors, or CKD; stage 4: clinical CVD, including coronary heart disease (CHD), HF, stroke, peripheral artery disease (PAD), and atrial fibrillation (AF) among individuals with excess/dysfunctional adiposity, other metabolic risk factors, or CKD [1].

The exclusion criteria included: (1) patients with less than two estimated glomerular filtration rate (eGFR) measurements during the follow-up period; (2) patients with kidney failure at baseline, including those with eGFR < 15 mL/min/1.73 m^2^, or with kidney replacement therapy (including maintenance hemodialysis, peritoneal dialysis, or kidney transplantation). (3) patients with acute kidney injury.

This study has been approved by the Ethics Committee of Peking University Third Hospital (IRB00006761-M2024424). Owing to the retrospective nature of the study, the requirement for written informed consent was waived.

### Data collection

All clinical data were extracted from the electronic health records. The index date was defined as the date of the first hospitalization during the study period that met the CKM stage 4 diagnostic criteria. All baseline variables were collected at the time of the index hospitalization. For laboratory values with multiple measurements during the index hospitalization, we used the first available measurement within 24 h of admission as the baseline value to reflect the patient’s initial clinical status. Follow-up began with the patient’s discharge time of the first hospitalization and continued until the earliest occurrence of the study outcome, death, or the end of the study period.

Demographic characteristics included age, sex, and smoking status. Physical examination data included BMI, WC, systolic blood pressure (SBP), diastolic blood pressure (DBP), and heart rate. Comorbidities included hypertension, DM, hyperlipidemia, MetS, CHD, stroke, AF, HF, and PAD. Medication history included the usage of insulin, renin-angiotensin-aldosterone system (RAAS) inhibitors, antiplatelet drugs, and statins. Laboratory examination data included white blood cell (WBC) counts, neutrophil counts, monocyte counts, lymphocyte counts, platelet counts, hemoglobin (HGB), serum creatinine (SCr), blood urea nitrogen (BUN), serum uric acid (UA), eGFR, urine protein (0–4+), urinary albumin-to-creatinine ratio (UACR), serum total protein, serum albumin (ALB), serum fibrinogen, alanine aminotransferase (ALT), aspartate aminotransferase (AST), total bilirubin, total cholesterol (TC), TG, high-density lipoprotein cholesterol (HDL-C), low-density lipoprotein cholesterol (LDL-C), FBG, HbA1c, and N-terminal pro-B-type brain natriuretic peptide (NT-proBNP). The imaging examination data included left ventricular ejection fraction (LVEF) and left ventricular end-diastolic diameter (LVEDD). The eGFR was calculated using the Chronic Kidney Disease Epidemiology Collaboration 2021 race-free equation [13]. Scr was measured using the sarcosine oxidase enzymatic method.

### Study outcomes

The primary endpoint was the subsequent deterioration of kidney function. The deterioration of kidney function was assessed based on eGFR derived from SCr measurements, identifying cases with a sustained decline in eGFR equal to or more than 40% or initiation of kidney replacement therapy [13, 14]. A sustained ≥ 40% decline in eGFR from baseline was confirmed by at least two consecutive measurements at least 3 months apart. When a patient’s follow-up ends before experiencing the outcome of interest, the patient is censored. Censoring can happen for any of a number of reasons, including death, loss to follow-up, and end of study.

### Statistical analysis

Analyses were performed using R software (version 4.3.1). Continuous variables were presented as a mean (standard deviation) or a median (interquartile range), whereas categorical data were presented as a number (percentage). An unpaired, 2-tailed *t*-test or a Mann-Whitney U-test was used to test group differences for continuous variables, and the Pearson *χ*^*2*^ test was used for categorical variables. *P-*value < 0.05 (two-sided) was considered to be significant. Variables with missing values ≥ 10% were excluded, while variables with missing values < 10% were subjected to multiple imputations using the mice package in R software [15]. We used the univariate Cox regression to select the potential variables. Before the multiple Cox regression, we calculated the collinearity of the variables and removed the factors that existed in collinearity. Then, we selected variables for the Cox prediction by a backward stepwise multivariate Cox regression with a *P*-value threshold of 0.1. Then we used the least absolute shrinkage and selection operator (LASSO) regression for variable selection to ensure the simplicity of the model and minimize overfitting. We applied the one-standard-error rule (SE) in LASSO regression to select the most concise model. Subsequently, variables for the LASSO-Cox prediction model were further selected by a backward stepwise multivariate Cox regression with a *P*-value threshold of 0.05. Furthermore, we employed a greedy algorithm in combination with Random Survival Forests (RSF) to select variables and construct a machine learning prediction model. We randomly allocated the subjects in a 2:1 ratio into a development cohort and a validation cohort. During the variable selection process in the development cohort, we applied a resampling-based strategy using 5-fold cross-validation repeated 500 times, to avoid overfitting. Variables were sequentially incorporated into the model according to the median area under the receiver operating characteristic curve (AUC) obtained across the resampling procedures, with preference given to those yielding the highest median AUC. The Bootstrap method was used for internal validation of the prediction models. The receiver operating characteristic (ROC) curves were used to evaluate the discrimination of the prediction models, and calibration curves were used to assess the calibration of the prediction models. We used the inverse probability of censoring weighting (IPCW) method to calculate time-dependent AUC. The analysis was implemented using the IPCW method in the timeROC package in R. The decision curve analysis (DCA) was used to evaluate the clinical utility of the predictive models, and the Shapley Additive explanations (SHAP) technique was used to interpret the machine learning model. We compared the newly established prediction model with the existing 4-variable Kidney Failure Risk Equation (KFRE) in the subset of participants with data on UACR. The net reclassification improvement (NRI) and integrated discrimination improvement (IDI) were also used to evaluate the discriminatory ability of the models.

## Results

### Baseline characteristics of the study population

**Fig. 1:**
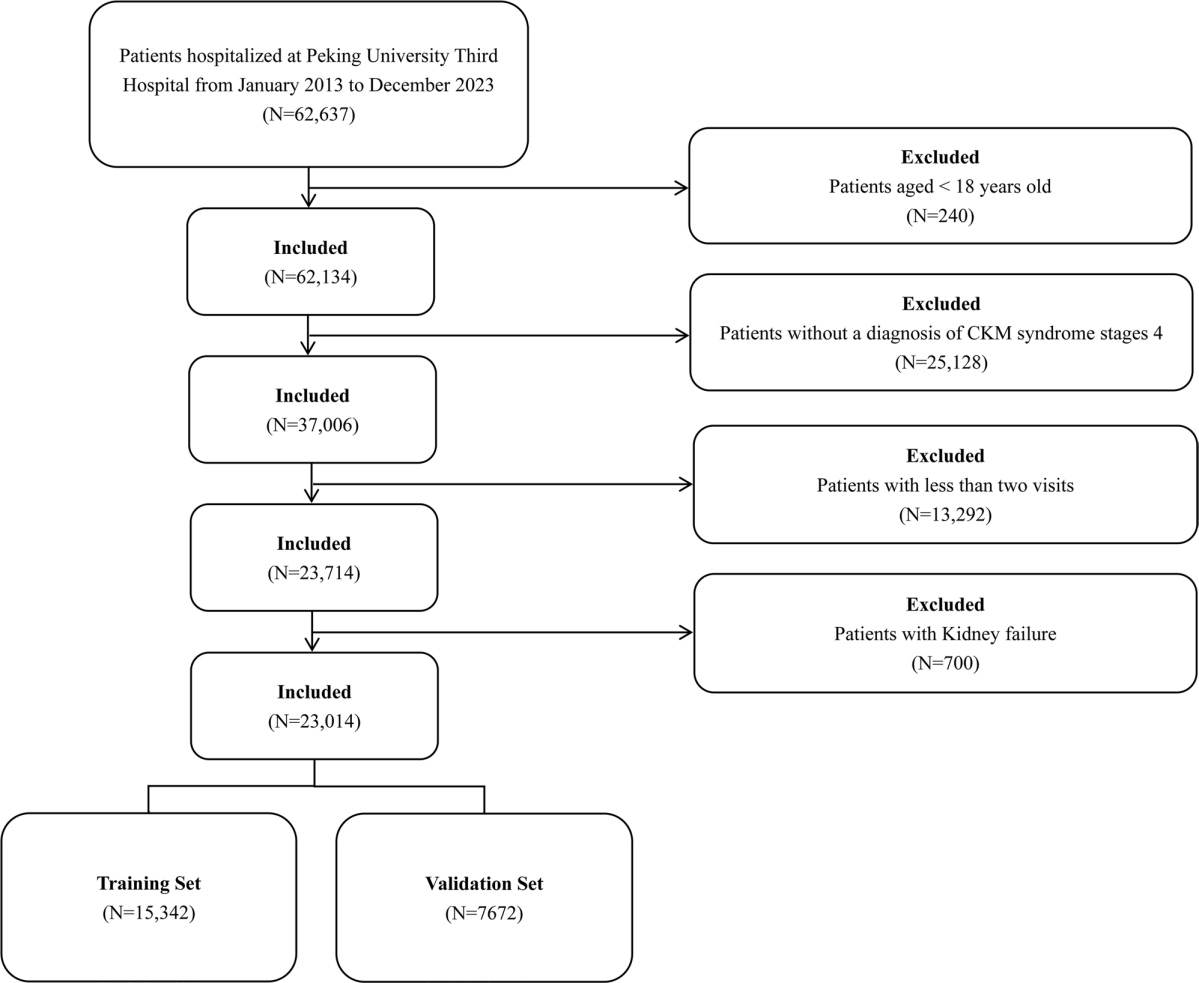
The flowchart for the selection of patients

From January 2013 to December 2023, 62,637 patients were screened for eligibility. After exclusions, the final analysis included 23,014 subjects who were randomly divided into two cohorts at a ratio of 2:1, comprising the training cohort (*n* = 15,342) and validation cohort (*n* = 7,672). The flowchart for the selection of patients in the derivation and the validation cohorts is shown in Fig. 1. The baseline clinical characteristics and laboratory parameters of the derivation cohort and validation cohort are shown in Table 1. Except for ALT, all variables showed no significant differences between the derivation and validation cohorts (*P* < 0.05). In the whole cohort, the average number of visits per patient was 4.6. The average follow-up time for the entire cohort was 34.65 months, with a median of 32.50 months (range 23.5 to 41.5 months). The average follow-up time was 34.75 months in training cohort and 34.48 months in validation cohort, respectively. In this study, 2714 patients experienced the outcome, 129 patients died, and 20,171 patients reached the end of the study without experiencing the outcome of interest. For patients who reached the outcome, died or without experiencing the outcome of interest, the median follow-up time was 20.8 months,17.3 months and 35.1 months, respectively. 1,772 outcome events (11.6%) occurred in the training cohort, while 942 outcome events (12.3%) were observed in the validation cohort. The number and proportion of patients experiencing a 40% decline in kidney function in the first, second, and third years are presented in Table S1. We also analyzed the clinical characteristics of patients with HF (See in Table S2).

**Table 1:** Baseline characteristic of the study population in derivation and validations

Variables	All patients (*n* = 23,014)	Derivation cohort (*n* = 15,342)	Validation cohort (*n* = 7,672)	*P*-value
**Demographic characteristics**				
Age (year)	66 (58, 75)	66 (58, 75)	66 (58, 75)	0.95
Sex (male, %)	15,430 (67.05)	10,264 (66.90)	5166 (67.34)	0.52
Smoking status				0.37
Nonsmoker	12,073 (52.46)	8087 (52.71)	3986 (51.96)	
Former smoker	9147 (39.75)	6049 (39.43)	3098 (40.38)	
Current smoker	1794 (7.80)	1206 (7.86)	588 (7.66)	
**Physical examination data**				
BMI (kg/m^2^)	24.91 (22.77, 27.28)	24.86 (22.64, 27.23)	24.96 (22.84, 27.32)	0.31
SBP (mmHg)	135(122, 147)	135 (122, 147)	135 (122, 147)	0.98
DBP (mmHg)	78 (70, 85)	78 (70, 85)	78 (70, 85)	0.96
Heart rate (bpm)	72 (66, 80)	72(66, 80)	72 (66, 80)	0.97
**Comorbidities**				
Hypertension (n, %)	16,247 (70.60)	10,871 (70.86)	5376 (70.07)	0.22
DM (n, %)	9005 (39.13)	5996 (39.08)	3009 (39.22)	0.85
Hyperlipidemia (n, %)	22,257 (96.71)	14,834 (96.69)	7423 (96.75)	0.82
MetS (n, %)	9925 (43.13)	6624 (43.18)	3301 (43.03)	0.84
CHD (n, %)	16,002 (69.53)	10,688 (69.66)	5314 (69.26)	0.54
Stroke (n, %)	7720 (33.54)	5179 (33.76)	2541 (33.12)	0.34
AF (n, %)	3003 (13.05)	1980 (12.91)	1023 (13.33)	0.37
HF (n, %)	3627 (15.76)	2413 (15.73)	1214 (15.82)	0.87
PAD (n, %)	1240 (5.39)	813 (5.30)	427 (5.57)	0.42
**Medication history**				
Insulin (n, %)	2116 (9.19)	1398 (9.11)	718 (9.36)	0.56
RAAS inhibitors (n, %)	9970 (43.32)	6606 (43.06)	3364 (43.85)	0.26
Antiplatelet drugs (n, %)	17,711 (76.96)	11,846 (77.21)	5865 (76.45)	0.20
Statins (n, %)	19,102 (83.00)	12,759 (83.16)	6343 (82.68)	0.36
**Laboratory examination data**				
WBC (×10^9^/L)	6.69 (5.52, 8.29)	6.70 (5.51, 8.32)	6.68 (5.49, 8.27)	0.48
Neutrophil (×10^9^/L)	4.24 (3.28, 5.51)	4.25 (3.28, 5.52)	4.24 (3.27, 5.51)	0.87
Monocytes (×10^9^/L)	0.42 (0.32, 0.54)	0.42 (0.32, 0.55)	0.42 (0.32, 0.54)	0.92
Lymphocyte (×10^9^/L)	1.68 (1.30, 2.15)	1.68 (1.29, 2.15)	1.69 (1.30, 2.16)	0.42
Platelet (×10^9^/L)	205 (167, 240)	205 (166, 240)	205 (167, 240)	0.91
HGB (g/L)	137 (126, 149)	137 (126, 149)	136 (125, 149)	0.62
SCr (µmol/L)	79 (68, 91)	79 (68, 91)	80 (69, 92)	0.1
BUN (mmol/L)	5.5 (4.5, 6.8)	5.4 (4.4, 6.8)	5.5 (4.5, 6.8)	0.74
Serum uric acid (µmol/L)	337 (278, 404)	338 (279, 404)	337 (278, 404)	0.46
eGFR (mL/min/1.73 m^2^)	82.57 (68.47, 95.94)	82.56 (68.46, 96.94)	82.58 (68.47, 94.96)	0.19
Urine protein				0.17
0 - ±	20,531 (89.21)	13,724 (89.45)	6807 (88.73)	
1 + − 2+	2021 (8.78)	1309 (8.53)	712 (9.28)	
3 + − 4+	462 (2.01)	309 (2.01)	153 (1.99)	
Serum total protein (g/L)	65 (61, 69)	65 (61, 68)	65 (61,69)	0.91
Serum ALB (g/L)	39.9 (37.4, 42.7)	39.9 (37.3, 42.7)	40.0 (37.4, 42.8)	0.15
Fibrinogen (g/L)	3.07 (2.68, 3.58)	3.08 (2.71, 3.60)	3.07 (2.68, 3.56)	0.23
ALT (U/L)	18 (13, 27)	16 (11, 25)	18 (13, 28)	< 0.01
AST (U/L)	21 (17, 29)	22 (17, 30)	21 (17, 29)	0.42
Total bilirubin (µmol/L)	13.3 (10.3, 17.2)	13.2 (10.2, 17.2)	13.3 (10.4, 17.2)	0.78
TC (mmol/L)	4.04 (3.41, 4.78)	4.05 (3.42, 4.79)	4.03 (3.40, 4.78)	0.15
TG (mmol/L)	1.41 (1.02, 1.97)	1.40 (1.01, 1.97)	1.42 (1.03, 1.98)	0.18
HDL-C (mmol/L)	0.97 (0.83, 1.15)	0.97 (0.82, 1.15)	0.97 (0.83, 1.15)	0.92
LDL-C (mmol/L)	2.40 (1.86, 3.05)	2.39 (1.84, 3.05)	2.41 (1.86, 3.06)	0.38
FBG (mmol/L)	5.6 (4.9, 6.9)	5.7 (4.9, 7.0)	5.6 (4.9, 6.9)	0.71
HbA1c (%)	6.1 (5.7, 7.1)	6.1 (5.7, 7.0)	6.1(5.7, 7.1)	0.98
NT-proBNP (pg/mL)	214.4 (75.9, 824.0)	214.3 (75.7, 824.0)	214.4 (76.0, 824.1)	0.77
**Imaging examination data**				
LVEF (%)	68 (60, 72)	68 (60, 71)	68 (60, 72)	0.92
LVEDD (mm)	48 (45.0, 51.2)	48 (45.0, 51.3)	48 (45.0, 51.1)	0.83

Abbreviations: BMI, body mass index; SBP, systolic blood pressure; DBP, diastolic blood pressure; DM, diabetes mellitus; MetS, metabolic syndrome; CHD, coronary heart disease; AF, atrial fibrillation; HF, heart failure; PAD, peripheral artery disease; RAAS, renin-angiotensin-aldosterone system; WBC, white bllod cell; HGB, hemoglobulin; SCr, serum creatinine; BUN, blood urea nitrogen; eGFR, estimated glomerular filtration rate; ALB, albumin; ALT, alanine aminotransferase; AST, aspartate aminotransferase; TC, total cholesterol; TG, triglyceride; HDL-C, high-density lipoprotein cholesterol; LDL-C, low-density lipoprotein cholesterol; FBG, fasting blood glucose; HbA1c, glycosylated hemoglobin; NT-proBNP, N-terminal pro-B-type brain natriuretic peptide; LVEF, left ventricular ejection fraction; LVEDD, left ventricular end-diastolic diameter

Missing data: Variables Scr, eGFR, BUN, and serum uric acid had 2.1% missing values. urine protein (dipstick) had 1.4% missing data. Hemoglobin had a missing rate of 2.6%. Serum total protein and serum ALB had a missing rate of 3.7%. BNP had a missing rate of 6.1%. LVEF had a missing rate of 7.4%. Variables with missing values ≥ 10% were excluded, while variables with missing values < 10% were subjected to multiple imputations using the mice package in R software

### Selection of predictive factors

#### Univariable and multivariable Cox regression analysis

A total of 30 variables were significantly associated with kidney function deterioration (*P* < 0.05). After the collinearity analysis (Table S3), significant variables including age, SBP, hypertension, DM, HF, usage of RAAS inhibitors, usage of antiplatelet drugs, HGB, BUN, urine protein, serum ALB, serum fibrinogen, TG, HbA1c, and LVEF were included in the multivariate Cox regression models (*P* < 0.05) (See in Table S4). Given that death may act as a competing event for the kidney outcome, we conducted an analysis using the Fine-Gray model and compared the variable effects with those from the Cox model. The results suggest that treating death as a competing event did not affect the consistency of overall conclusions (See in Table S5).

#### LASSO-Cox regression analysis

To mitigate overfitting concerns and address multicollinearity among variables, we employed LASSO-Cox regression. First, the LASSO regression screened 18 independent variables (Figure S1). Subsequently, multivariate Cox regression refined this selection, ultimately identifying 15 predictive factors (*P* < 0.05) including age, DM, hypertension, AF, HF, use of antiplatelet drugs, SBP, HGB, fibrinogen, TG, HbA1c, serum ALB, UA, urinary protein, and LVEF (Table S6).

#### Random survival forest analysis

An RSF method (ntree = 800) was used to calculate the Mean Decrease Accuracy (MDA) of each variable. The results showed that LVEF ranked first in importance, followed by NT-proBNP, urine protein, BUN, age, and ALB. The least important target variable was stroke. By using the greedy algorithm along with RSF for selecting variables, eight variables including NT-proBNP, serum ALB, LVEF, HGB, age, BUN, urine protein and Scr were selected for RSF model constructing. The variable selection process of the RSF model is shown in Figure S2.

### Model development and performance evaluation

**Fig. 2:**
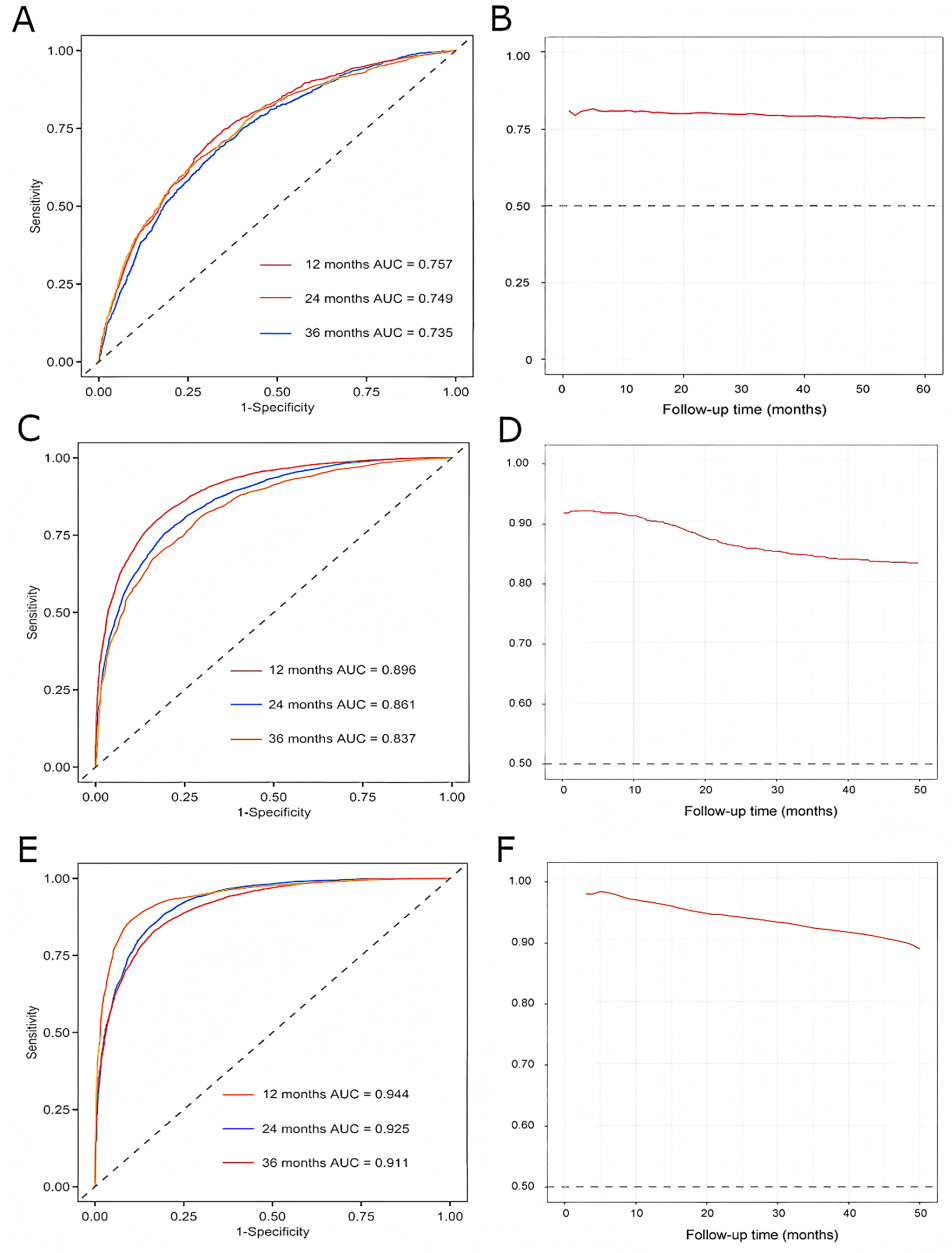
Discrimination of the Cox model, LASSO-Cox model and the RSF model in the derivation cohort. The discrimination of the Cox model in the derivation cohort based on ROC analysis during a follow-up time of 12 months, 24 months and 36 months; **B**. The time-dependent ROC curve of the Cox model in the derivation cohort; **C**. The discrimination of the LASSO-Cox model in the derivation cohort based on ROC analysis during a follow-up time of 12 months, 24 months and 36 months; **D**. The time-dependent ROC curve of the LASSO-Cox model in the derivation cohort; **E**. The discrimination of the RSF model in the derivation cohort based on ROC analysis during a follow-up time of 12 months, 24 months and 36 months; **F**. The time-dependent ROC curve of the RSF model in the derivation cohort. Abbreviations: LASSO: least absolute shrinkage and selection operator; RSF: random survival forest; ROC: receiver operating characteristic; AUC, area under curve

**Fig. 3:**
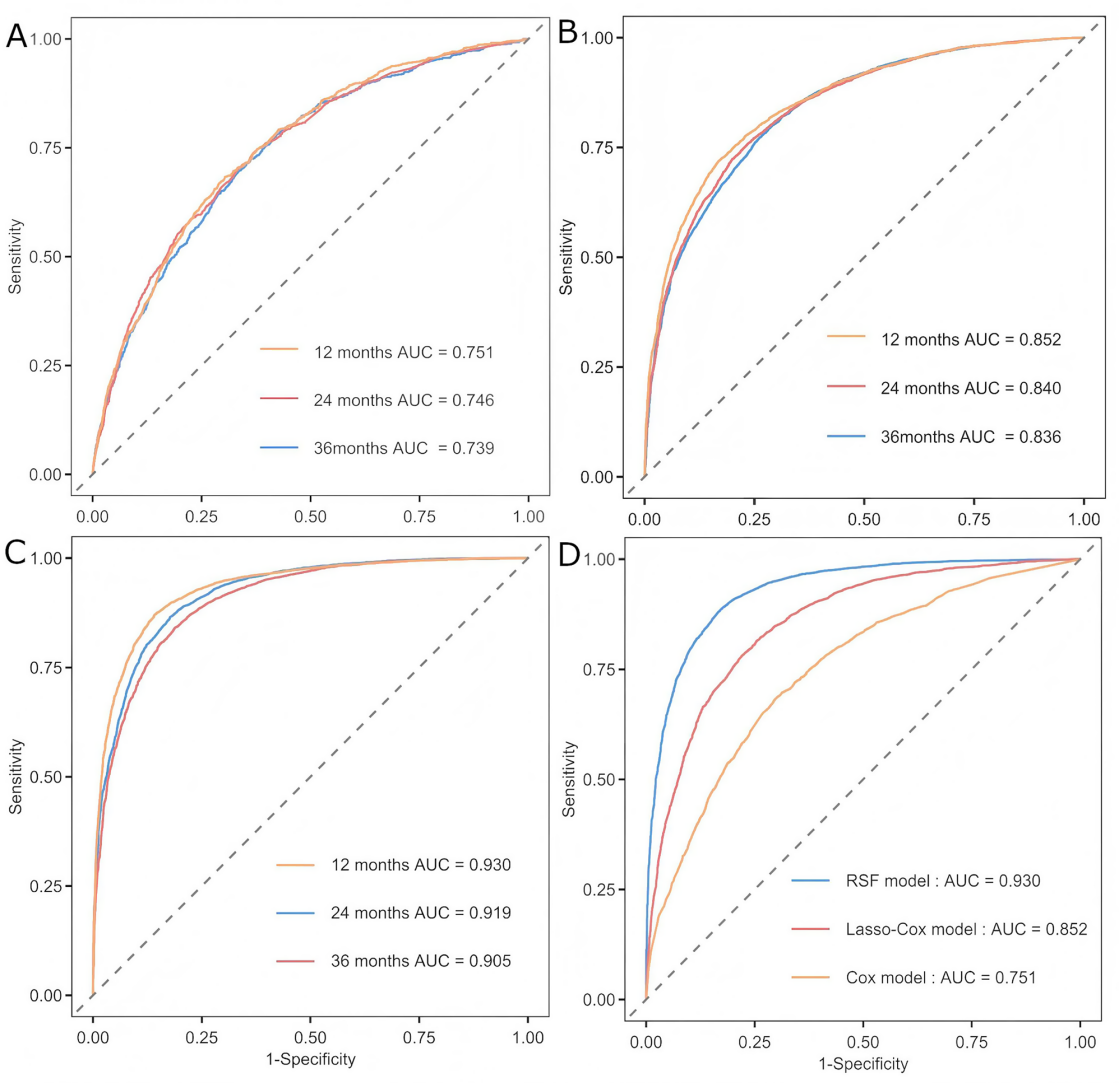
Discrimination of the Cox model, LASSO-Cox model and the RSF model in the internal validation cohort. The discrimination of the Cox model in the internal validation cohort based on ROC analysis during a follow-up time of 12 months, 24 months and 36 months; **B**. The discrimination of the LASSO-Cox model in the internal validation cohort based on ROC analysis during a follow-up time of 12 months, 24 months and 36 months; **C**. The discrimination of the RSF model in the internal validation cohort based on ROC analysis during a follow-up time of 12 months, 24 months and 36 months. **D**. The discrimination of the Cox model, LASSO-Cox model and the RSF model during a follow-up period of 12 months (Delong test, *P* < 0.001). Abbreviations: LASSO: least absolute shrinkage and selection operator; RSF: random survival forest; ROC: receiver operating characteristic

**Fig. 4:**
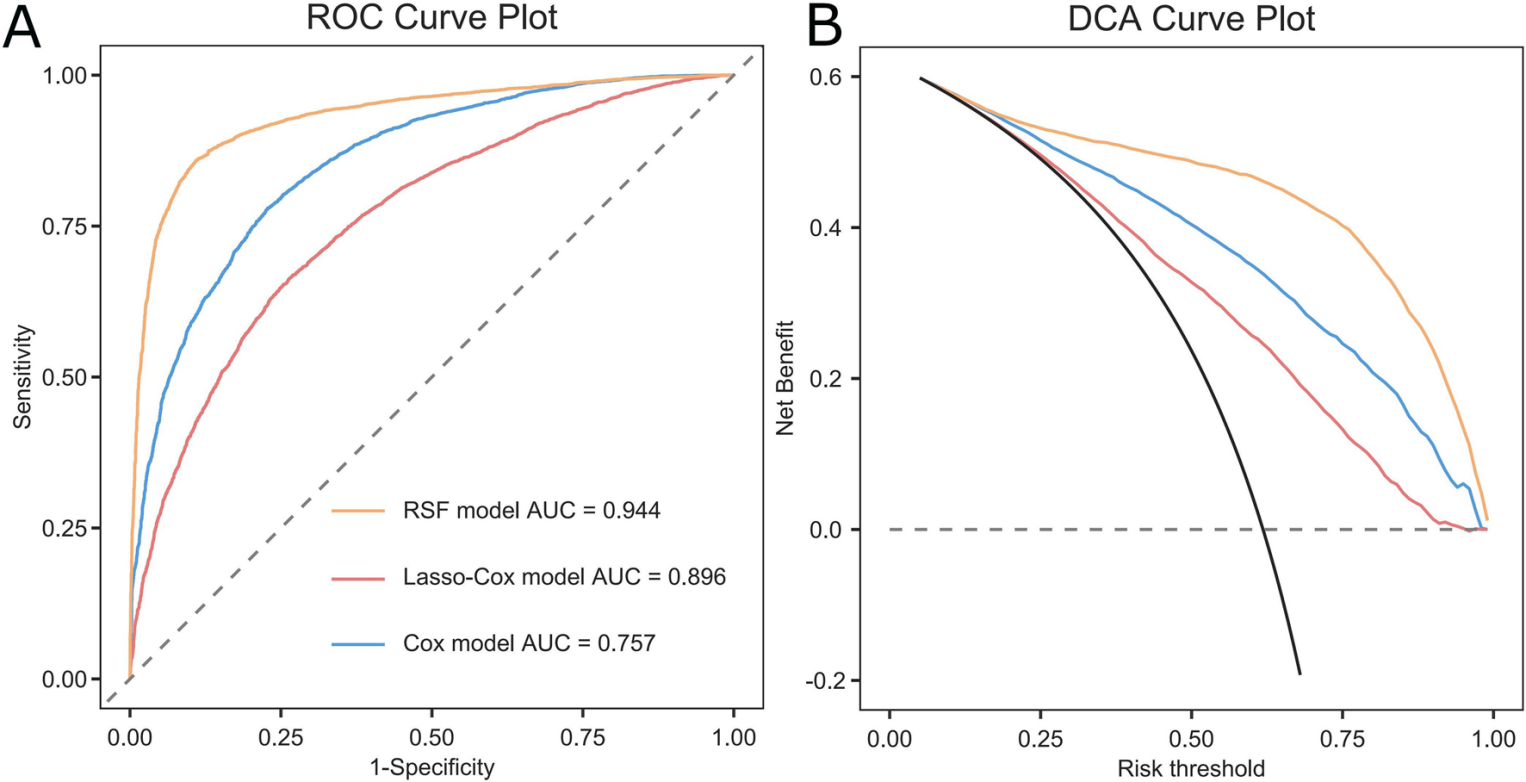
Predictive performance of the three models in the derivation cohort at 12 months. (**A**) Receiver operating characteristic, (**B**) Decision curve analysis. Abbreviations: AUC: area under the curve; RSF: random survival forest

The performances for the Cox model, LASSO-Cox model, and RSF model were reported in Table 2. Among the three models, the RSF model achieved the highest AUC and C-index in both the derivation and validation cohorts (derivation set: AUC 0.944, C-index 0.914; validation set: AUC 0.930; C-index 0.906). The risks of outcome events at 12-, 24-, and 36-month intervals in both the derivation and validation cohorts for the three models are depicted in Figs. 2 and 3, respectively. The number of patients at risk and the censoring status at each prediction time point (12, 24, and 36 months) of the validation cohort are shown in Table S7. In the derivation cohort, the discrimination of the RSF model was significantly higher than that of the Cox model and LASSO-Cox model. And the DCA at 12 months demonstrated that the RSF model had a superior net benefit compared to both the Cox model and the LASSO-Cox model (Fig. 4). In general, among the three models evaluated, the RSF model demonstrated clear superiority in discriminative ability and exhibited good clinical utility. There were no significant differences between the observed and predicted outcome events in the validation set (Figure S3 and Figure S4).

**Table 2:** Performances of three models in the derivation and validation cohorts during a follow-up time of 12 months

	Cox model	Lasso-Cox model	RSF model
AUC			
training cohort	0.757(95%CI 0.739–0.775)	0.896(95%CI 0.890–0.902)	0.944(95%CI 0.939–0.948)
Internal validation cohort	0.751(95%CI 0.734–0.767)	0.852(95%CI 0.845–0.859)	0.930(95%CI 0.925–0.934)
C index			
training cohort	0.704(95%CI 0.697–0.711)	0.840(95%CI 0.834–0.846)	0.914(95%CI 0.908–0.919)
Internal validation cohort	0.693(95%CI 0.683–0.703)	0.837(95%CI 0.829–0.845)	0.906(95%CI 0.901–0.912)

Abbreviations: AUC: Area Under the ROC Curve; LASSO: least absolute shrinkage and selection operator; RSF: Random Survival Forests

### Temporal validation

We use a temporal validation strategy for model validation. Based on the admission time of each patient, 12,054 patients admitted before December 31, 2018, were used as the derivation cohort, and 10,960 patients admitted after December 31, 2018, were used as the validation cohort. The performances for the Cox model, LASSO-Cox model, and RSF model were reported in Table 3. Among the three models, the RSF model achieved the highest AUC and C-index in both the derivation and validation cohorts (derivation set: AUC 0.937, C-index 0.910; validation set: AUC 0.917; C-index 0.898).

**Table 3:** Performances of three models in the derivation and temporal validation cohorts during a follow-up time of 12 months

	Cox model	Lasso-Cox model	RSF model
AUC			
Derivation cohort	0.748 (95%CI 0.731–0.765)	0.811 (95%CI 0.791–0.831)	0.937 (95%CI 0.932–0.943)
Temporal validation cohort	0.734 (95%CI 0.717–0.751)	0.807 (95%CI 0.787–0.828)	0.917 (95%CI 0.911–0.924)
C index			
Derivation cohort	0.687 (95%CI 0.681–0.693)	0.798 (95%CI 0.776–0.820)	0.910 (95%CI 0.905–0.916)
Temporal validation cohort	0.671 (95%CI 0.664–0.678)	0.792 (95%CI 0.771–0.813)	0.898 (95%CI 0.892–0.904)

Abbreviations: AUC: Area Under the ROC Curve; LASSO: least absolute shrinkage and selection operator; RSF: Random Survival Forests

### Model explanation

**Fig. 5:**
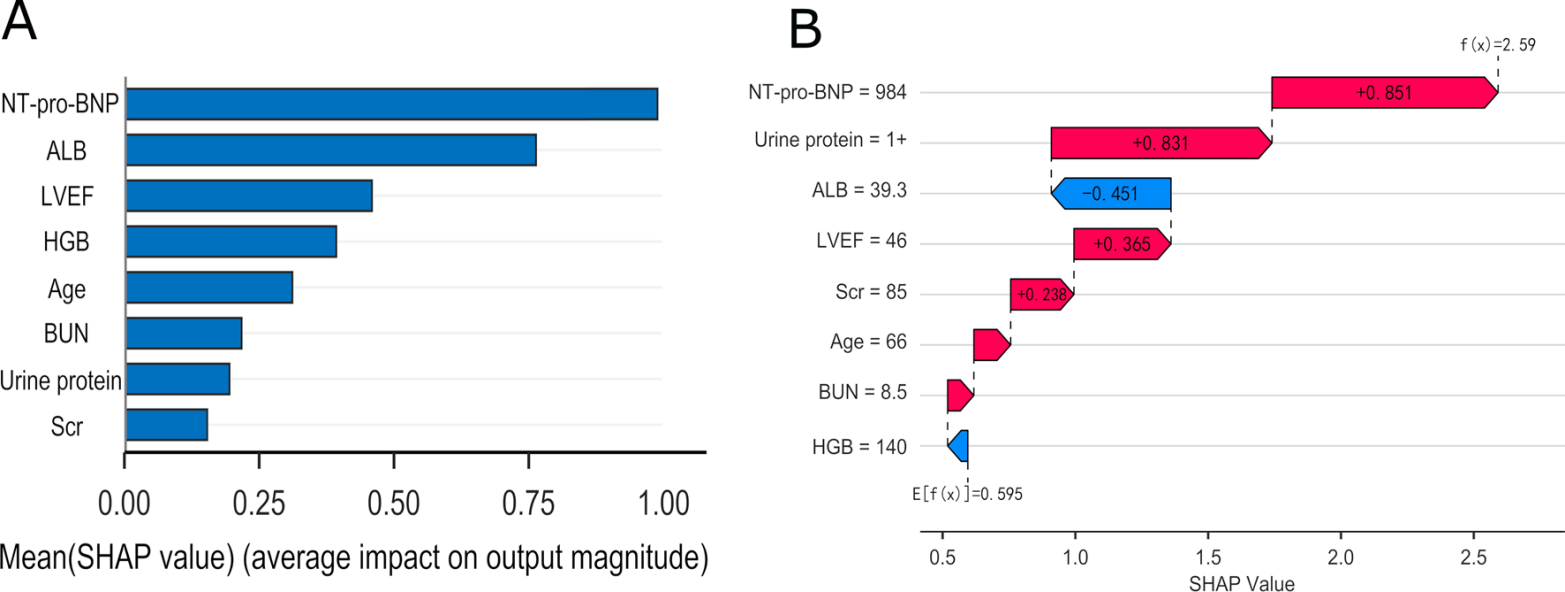
Model explanation using the SHAP method. (**A**) Importance matrix plot of the RSF model. (**B**) The SHAP waterfall plot of RSF model, explaining outcome event risk for an individual patient. Variables in the red arrow mean the impact values are positive while blue means negative. E[f(x)] represents the baseline value, which is the model’s predicted evaluation for all patients. f(x) denotes the final prediction made by the model for that specific patient. A higher F(x) than E[f(x)] indicates that the patient’s predicted risk exceeds the average threshold. Abbreviations: NT-proBNP, N-terminal pro-B-type brain natriuretic peptide; LVEF, left ventricular ejection fraction; ALB, albumin; HGB, hemoglobulin; BUN, blood urea nitrogen

To elucidate feature contributions and interpret the model’s predictions, we employed SHAP analysis to quantify the relative importance of each predictor. From a global perspective, the SHAP summary plot (Fig. 5A) displays the mean absolute SHAP value for each feature, ranking them according to their overall contribution to predicting kidney function progression in patients with stage 4 CKM syndrome. NT-proBNP, ALB, and LVEF were identified as the top three most influential predictors in the RSF model. These features exerted a notable influence on the model output, underscoring their importance in clinical risk stratification. At the individual level, SHAP waterfall plots were utilized to deconstruct the model’s prediction for specific patients (Fig. 5B). For instance, in a representative patient, predictors including NT-proBNP = 984 pg/mL, proteinuria (1+), serum albumin = 39.3 g/L, and LVEF = 46% substantially shifted the risk prediction, with additional contributions from age, BUN, and HGB.

### Convenient application for clinical utility

To enhance clinical utility, the RSF prediction model was implemented as an interactive web application. People can input 8 required clinical features: NT-proBNP, serum ALB, LVEF, HGB, age, BUN, urine protein and SCr to obtain an automated, patient-specific kidney function progression risk estimation for individuals with stage 4 CKM syndrome.

### Model comparison

To evaluate the value of the newly developed clinical prediction model in patients with stage 4 CKM syndrome, we compared the RSF model with the classic 4-variable Kidney Failure Risk Equation (KFRE) (Table S8). Due to a high rate of missing values for the UACR in the main database, a subset with complete UACR data was established for this comparison. This subset comprised data from 6,911 patients. Within this subset, we compared the performance of the RSF model and the KFRE model in predicting the risk of kidney function progression. For predicting the 2-year risk of disease progression, the RSF model achieved a time-dependent AUROC of 0.921 (95% CI, 0.912–0.929), which significantly outperformed (*P* < 0.001) the KFRE model (AUROC, 0.890; 95% CI, 0.882–0.899). Furthermore, compared to the KFRE model, the RSF model demonstrated a statistically significant improvement in reclassification performance, as assessed by the integrated discrimination improvement (IDI: 0.08) and net reclassification improvement (NRI: 0.22).

## Discussion

Our study investigates predictors for the deterioration of kidney function and has successfully developed interpretable predictive models based on clinical indicators for the deterioration of kidney function in patients with stage 4 CKM syndrome.

Although prediction models for kidney function deterioration have been developed in many studies, these models have their own advantages and limitations. For example, the CKD-PC equation was constructed based on an ultra-large-scale database, but the proportion of data from developing countries is relatively small, and mainly incorporates common biomarkers [8, 16]. KidneyIntelX enhanced predictive capability by integrating novel biomarkers, yet its initial development and validation were based on a relatively small cohort [9, 10, 17]. Notably, in our present study, the RSF model demonstrates superior predictive performance (derivation set: AUC 0.942; validation set: AUC 0.927, in 12 months) and good calibration in both the derivation and validation cohorts than both Cox regression model and LASSO-Cox regression model. Moreover, The RSF model demonstrated significant net benefits in predicting the risk of kidney function deterioration at the 12-month time point, indicating substantial clinical utility for short-term decision-making. To enhance clinical utility, the RSF prediction model was implemented as an interactive web application. Based on this, future prospective research may provide more comprehensive information. The final selected RSF model included 8 variables: NT-proBNP, serum ALB, LVEF, HGB, age, BUN, urine protein and SCr. These variables are clinically accessible and cost-effective, demonstrating better feasibility in the practical application of predictive models. Notably, other than well-established predictors for kidney function progression such as proteinuria [18, 19], our study revealed that cardiac functional markers, particularly LVEF and NT-proBNP, exhibit good predictive value for kidney function decline in patients with stage 4 CKM syndrome. Reduced LVEF and elevated NT-proBNP levels have been consistently associated with kidney function decline in both CKD and non-CKD populations [20–23], but their value in stage 4 CKM patients is unclear. The elevations in NT-proBNP and decline in LVEF may not only reflect chronic neurohormonal activation, hemodynamic derangement and impaired cardiac function, but also underscore the important role of cardiorenal interactions in driving kidney function deterioration in patients with CKM syndrome [24, 25]. Incorporating these cardiac biomarkers into a kidney prognostic model underscores the integrative pathophysiology linking cardiac and kidney dysfunction highlighting the necessity of a multidisciplinary approach for managing complex cardiometabolic and kidney diseases. Conversely, the progression of kidney disease may also exacerbate cardiac dysfunction, reflecting the intricate bidirectional interplay between the heart and kidneys [26].

Due to the “black box” nature of machine learning prediction models, we employed the SHAP method to interpret the model’s predictions, which provided global and patient-level explanations of the predictive model. Accordingly, the top two most important predictive indicators are NT-proBNP and ALB. These results may indicate that clinicians should be advised that when managing patients with Stage 4 CKM, despite the complexity of their condition and the variability of clinical indicators, these two markers hold significant predictive value for subsequent kidney function deterioration and should be prioritized for reference. Furthermore, we developed a web-based risk calculator to assist clinicians in identifying patients with stage 4 CKM syndrome at high risk of kidney function progression, thereby facilitating clinical utility. This model demonstrates several methodological strengths, including its foundation in real-world evidence, specific focus on patients at CKM Stage 4, and utilization of routinely collected clinical parameters for prediction, thereby enhancing its translational relevance to clinical practice. While the model exhibits robust overall predictive performance, its single-center derivation introduces potential limitations such as overfitting, necessitating external validation to optimize generalizability. This tool is designed for clinical application in kidney function assessment among hospitalized late-stage patients.

Our study selected kidney function progression as the primary endpoint, comprising a sustained decrease in eGFR of ≥ 40% or the initiation of kidney replacement therapy. The currently established hard end points of kidney failure and doubling of serum creatinine level, equivalent to a 57% decline in eGFR, are late events in CKD, requiring large clinical trials with long follow-up. As part of a comprehensive evaluation of lesser declines in eGFR as alternative end points, a previous meta-analysis included 9,488 participants from 37 randomized controlled trials of CKD progression across 5 intervention types. They found that the ratio (95% CI) of the HR for the alternative to established end point for the 5 intervention types ranged from 0.91 (0.64–1.43) to 1.12 (0.89–1.40) for 40% eGFR decline and from 0.88 (0.63–1.39) to 1.15 (0.88–1.54) for 30% decline for the overall study duration, indicating consistency of treatment effects. These results provide some support for the use of lesser eGFR declines as a surrogate end point, with stronger support for the 40% than 30% decline [27]. An estimated GFR decline of 40% may be more broadly acceptable than a 30% decline across a wider range of baseline GFRs and patterns of treatment effects on GFR [14, 28, 29]. Previous study results also indicated that a relative eGFR decline of 40% or greater is strongly associated with an increased risk of kidney failure, cardiovascular outcomes and all-cause mortality [30]. Therefore, in our study, we chose a decline of 40% or greater in eGFR as one of the outcomes.

Our study has several limitations. First, the dataset originates from a single Chinese tertiary hospital; therefore, overfitting could exist and external validation to improve the model may be needed in future studies. Second, certain variables, such as UACR, serum calcium and serum phosphorus, were not included in the statistical analysis due to a high proportion of missing values (> 10%). Consequently, detailed information on urinary protein and chronic kidney disease-mineral and bone disorder (CKD-MBD) indicators is currently lacking. Third, the monocentric, retrospective design without external validation is a significant limitation, particularly for identifying and ranking predictive factors. Further multi-center study is needed to confirm the result from the present study. Moreover, the generalizability of the findings is constrained by the using of a monoethnic study population and by the differing BMI and waist-circumference criteria in the CKM definitions for Asian versus non-Asian populations. Weighed against all above limitations, by using a large sample size, our study successfully established interpretable predictive model for predicting kidney function progression in stage 4 CKD patients and, using interpretable methods, identified that key factors especially NT-proBNP and decline in LVEF were associated with kidney function changes, demonstrating significant clinical value of these cardiac indicators and highlighting the necessity for multidisciplinary collaboration between cardiology and nephrology in stage 4 CKM patients’ management.

## Conclusions

This study explored factors associated with patents kidney function decline in CKM syndrome stage 4 patients and established validated, explainable and easy to use clinical risk prediction models, which may help guide patient risk stratification and patients’ management.

## Supplementary Information

Below is the link to the electronic supplementary material.

Supplementary Material 1

## Data Availability

The datasets used and/or analysed during the current study are available from the corresponding author on reasonable request.

## Abbreviations

CKM: Cardiovascular-kidney-metabolic
LASSO: Least absolute shrinkage and selection operator
AUC: Area under the curve
CI: Confidence interval
CKD: Chronic kidney disease
CVD: Cardiovascular disease
BMI: Body mass index
WC: Waist circumference
FBG: Fasting blood glucose
HbA1c: Glycosylated hemoglobin
TG: Triglyceride
DM: Diabetes mellitus
HF: Heart failure
CHD: Coronary heart disease
PAD: Peripheral artery disease
AF: Atrial fibrillation
GFR: Glomerular filtration rate
SBP: Systolic blood pressure
DBP: Diastolic blood pressure
RAAS: Renin-angiotensin-aldosterone system
WBC: White blood cell
HGB: Hemoglobulin
SCr: Serum creatinine
BUN: Blood urea nitrogen
UA: Uric acid
UACR: Urinary albumin-to-creatinine ratio
ALB: Albumin
ALT: Alanine aminotransferase
AST: Aspartate aminotransferase
TC: Total cholesterol
HDL-C: High-density lipoprotein cholesterol
LDL-C: Low-density lipoprotein cholesterol
NT-proBNP: N-terminal pro-B-type natriuretic peptide
LVEF: Left ventricular ejection fraction
LVEDD: Left ventricular end-diastolic diameter
SE: Standard error
RSF: Random survival forest
MDA: Mean decrease in accuracy
ROC: Receiver operating characteristic
DCA: Decision curve analysis
SHAP: Shapley additive explanations
NRI: Net reclassification
IDI: Integrated discrimination improvement
IPCW: Inverse probability of censoring weighting
MetS: Metabolic syndrome
CKD-MBD: Chronic kidney disease-mineral and bone disorder
