# Supplementary material for: Interpretable predictive model for deterioration of kidney function in patients with stage 4 cardiovascular-kidney-metabolic syndrome
Source: BMC Nephrol. 2026 Apr 20;27:348. doi: 10.1186/s12882-026-05001-0 (PMC13224402; doi:10.1186/s12882-026-05001-0)
Supplement: Supplementary file 1 — Supplementary Material 1 [file 12882_2026_5001_MOESM1_ESM.doc]

**Supplementary Materials**

**Supplementary Table of Contents**

1. **Figure S1.** Variable selection using the LASSO regression analysis with ten-fold cross-validation.
2. **Figure S2**. The variable selection process of the RSF model.
3. **Figure S3.** Calibration of the COX model and LASSO-Cox model and the RSF model in the internal validation cohort
4. **Figure S4**. Calibration of the COX model and the LASSO-Cox model and the RSF model in the internal validation cohort
5. **Table S1**. Annual Outcomes of Patients with a 40% decline in kidney Function in the derivation cohort
6. **Table S2**. Baseline Characteristics and Outcomes of Patients Stratified by History of Heart Failure
7. **Table S3**. Collinear analysis of potential risk factors
8. **Table S4**. Potential risk factors identified by Cox regression analysis
9. **Table S5**. Comparison of predictor effects between Cox model and Fine-Gray competing risk model for predicting kidney function progression
10. **Table S6.** The results of LASSO-Cox regression
11. **Table S7**. The number of at-risk individuals and censored individuals at each time point in the time-dependent ROC curve of the validation cohort
12. **Table S8**. Performance Comparison of RSF Model and KFRE Model
13. **The web address** for the web-based risk calculator based on the RSF model.

**
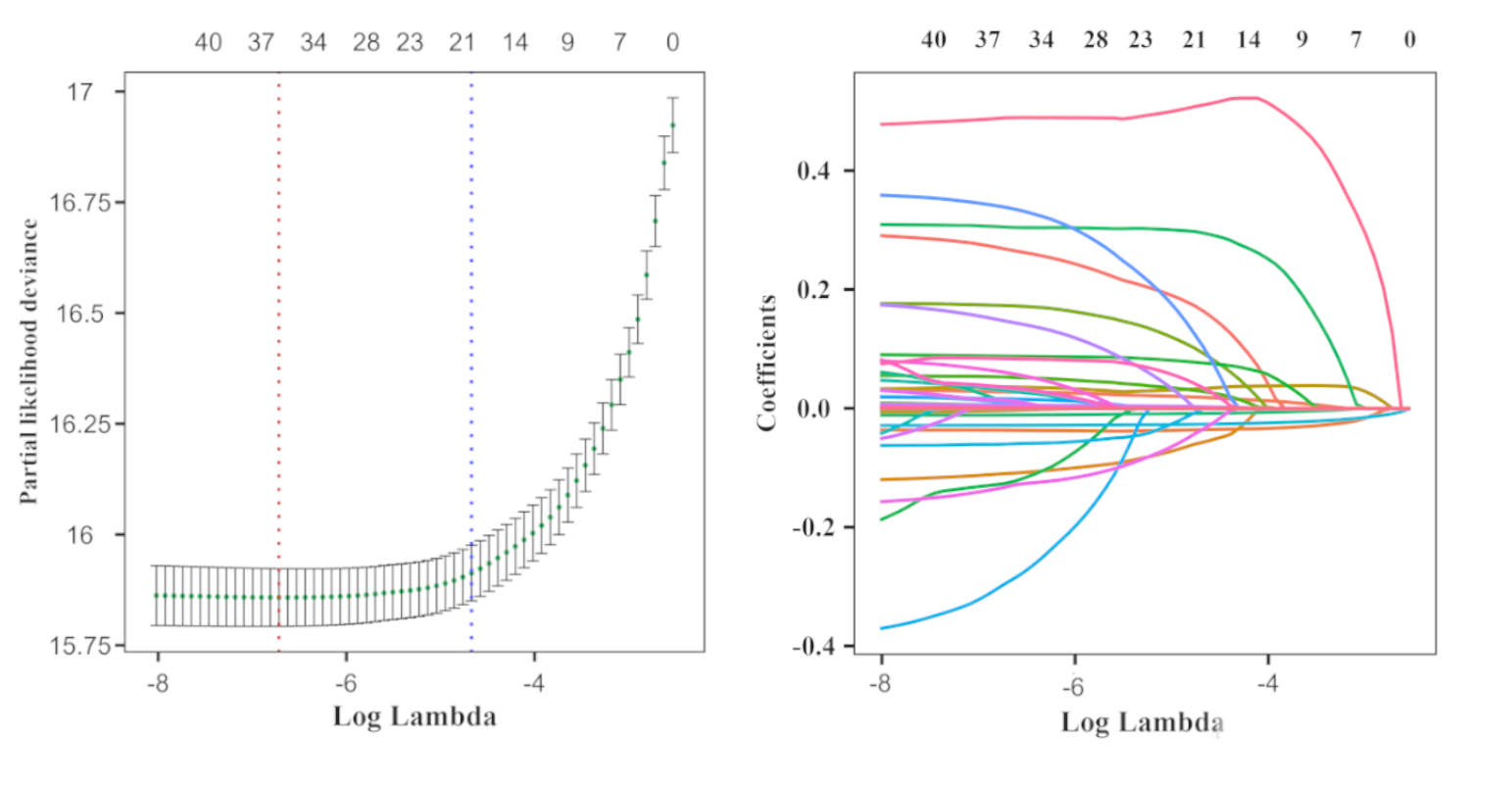
**

**Figure S1.** Variable selection using the LASSO regression analysis with ten-fold cross-validation.

1. Tuning parameter (λ) selection of deviance in the LASSO regression based on the minimum criteria (left dotted line) and the 1-SE criteria (right dotted line). B. A coefficient profile plot of 46 variables was created against the log (λ) sequence. In the present study, the predictor’s selection was according to the “one SE” criteria (right dotted line), where nonzero coefficients were selected.

**Abbreviations:** LASSO: least absolute shrinkage and selection operator.

**
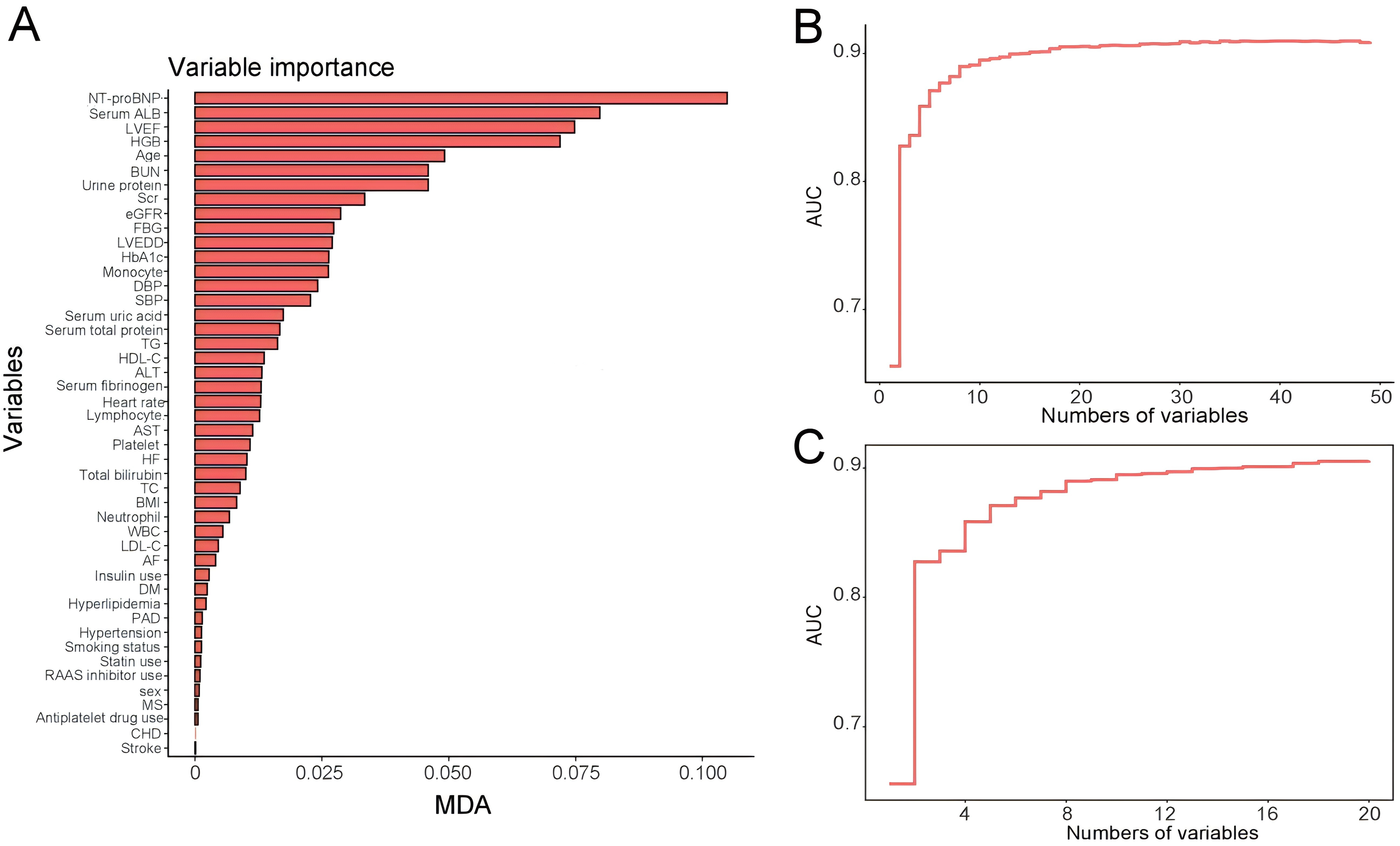
**

**Figure S2.** The variable selection process of the RSF model.

1. Variable importance of the candidate variables in the RSF model; B-C. Variable selection process using the “greedy algorithm” and the RSF method.

**Abbreviations:** LVEF, left ventricular ejection fraction; NT-proBNP, N-terminal pro-B-type brain natriuretic peptide; BUN, blood urea nitrogen; Scr, serum creatinine; ALB, albumin; HGB, hemoglobulin; eGFR，estimated glomerular filtration rate；FBG, fasting blood glucose; LVEDD, left ventricular end-diastolic diameter；HbA1c, glycosylated hemoglobin; DBP, diastolic blood pressure; SBP, systolic blood pressure; TG, triglyceride; HDL-C, high-density lipoprotein cholesterol; ALT, alanine aminotransferase; AST, aspartate aminotransferase; HF, heart failure; TC, total cholesterol; BMI, body mass index; WBC, white blood cell; LDL-C, low-density lipoprotein cholesterol; AF, atrial fibrillation; DM, diabetes mellitus; PAD, peripheral artery disease; MetS, metabolic syndrome; CHD, coronary heart disease.


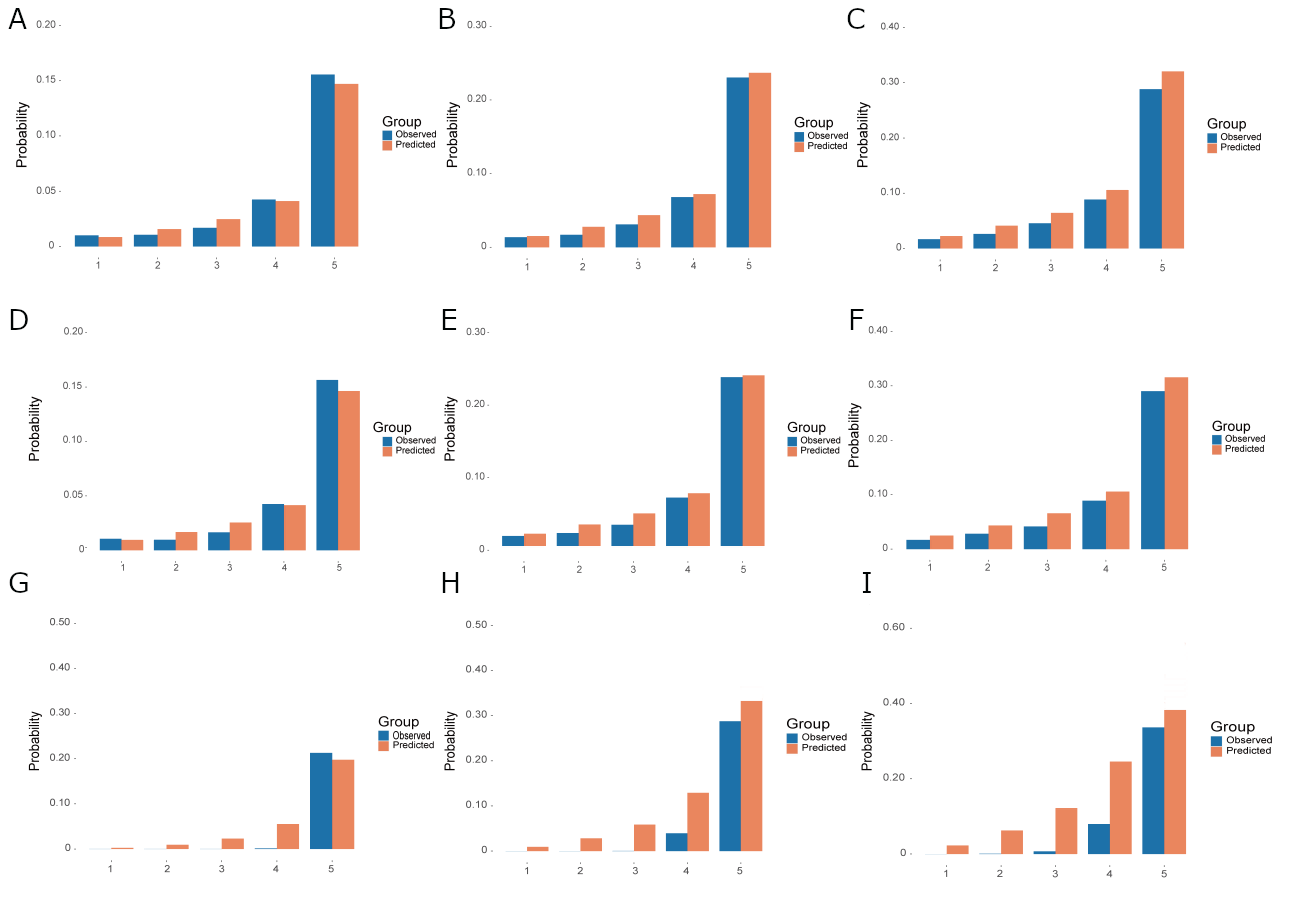
**Figure S3.** Calibration of the Cox model, LASSO-Cox model and the RSF model in the derivation cohort.

A-C. The calibration curve of the Cox model in the derivation cohort during a follow-up time of 12, 24, and 36 months, respectively; D-F. The calibration curve of the LASSO-Cox model the derivation cohort during a follow-up time of 12, 24, and 36 months; G-I.The calibration curve of the RSF model in the derivation cohort during a follow-up time of 12, 24, and 36 months.

**Abbreviations:** LASSO, least absolute shrinkage and selection operator; RSF, random survival forest.

**
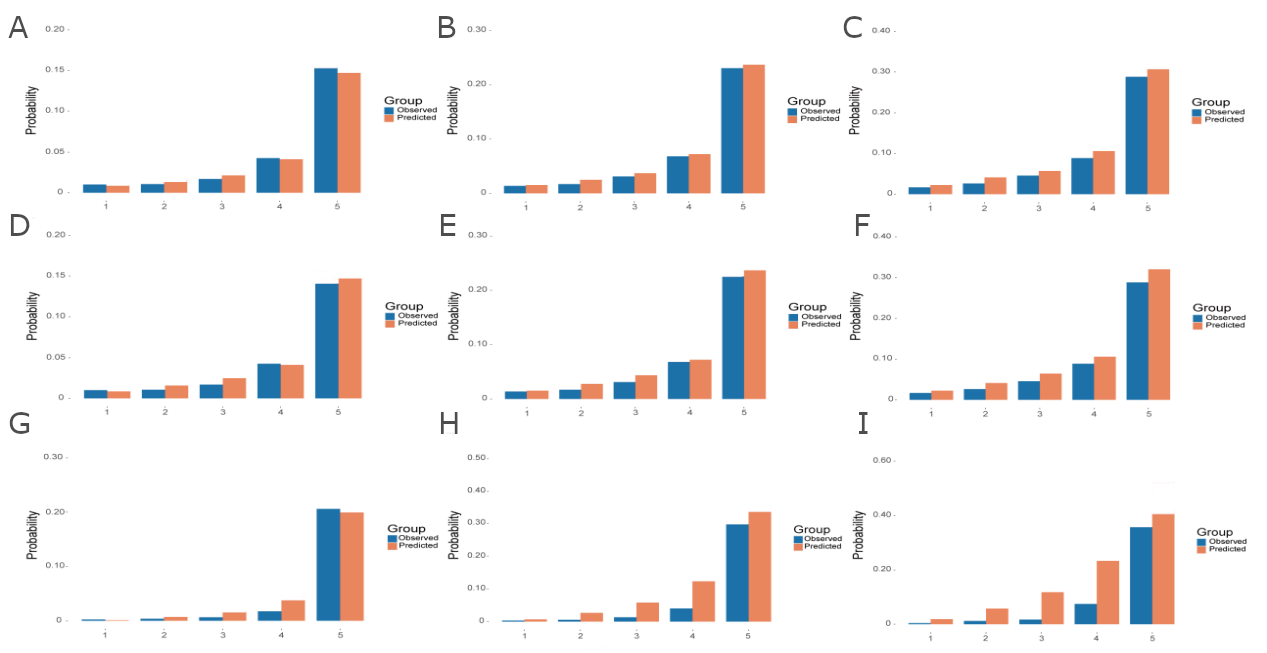
**

**Figure S4.** Calibration of the COX model and the LASSO-Cox model and the RSF model in the internal validation cohort

A-C. The calibration curve of the Cox model in the internal validation cohort during a follow-up time of 12, 24, and 36 months, respectively;

D-F.The calibration curve of the LASSO-Cox model in the internal validation cohort during a follow-up time of 12, 24, and 36 months, respectively;

G-I. The calibration curve of the RSF model in the internal validation cohort during a follow-up time of 12, 24, and 36 months, respectively.

**Abbreviations:** LASSO: least absolute shrinkage and selection operator; RSF: random survival forest.

**Table S1. Annual Outcomes of Patients with a 40% decline in kidney function in the derivation cohort**

| **Time Point**  **(Year)** | **Number of New Events**  **(n)** | **Cumulative Events**  **(n)** | **Cumulative Incidence, %**  **(95% CI)** |
| --- | --- | --- | --- |
| 1 | 196 | 196 | 1.28 (1.10 - 1.46) |
| 2 | 136 | 332 | 2.16 (1.93 - 2.39) |
| 3 | 144 | 476 | 3.10 (2.83 - 3.38) |

**Table S2. Baseline Characteristics and Outcomes of Patients Stratified by History of Heart Failure**

| **Variables** | **Patients with Heart Failure**  **(n=3,627)** | **Patients without Heart Failure**  **(n=19,387)** | ***P*-value** |
| --- | --- | --- | --- |
| **Demographic characteristics** | | | |
| Age (year) | 68 (59, 78) | 65 (57, 74) | <0.001 |
| Sex (male, %) | 2,450 (67.54%) | 13,202 (68.10%) | 0.499 |
| Smoking status |  |  | <0.001 |
| Nonsmoker | 2,043 (56.33%) | 10,030 (51.74%) |  |
| Former smoker | 1344 (37.06%) | 7803 (40.25%) |  |
| Current smoker | 240(6.62%) | 1554 (8.02%) |  |
| **Physical examination data** | | | |
| BMI (kg/m^2^) | 24.38 (22.03 - 27.06) | 24.98 (22.86 - 27.34) | | <0.001 | | --- | |
| SBP (mmHg) | 136 (124, 149) | 134 (122, 146) | <0.001 |
| DBP (mmHg) | 78 (70, 85) | 76 (68, 83) | <0.001 |
| Heart rate (bpm) | 75 (67, 80) | 72 (66, 80) | <0.001 |
| **Comorbidities** | | | |
| Hypertension (n, %) | 2,609 (71.93%) | 13,638 (70.35%) | 0.054 |
| DM (n, %) | 1,459 (40.23%) | 7,546 (38.92%) | 0.132 |
| Hyperlipidemia (n, %) | 3,433 (94.65%) | 18,824 (97.1%) | <0.001 |
| MS (n, %) | 1601 (44.14%) | 8,324 (42.94%) | 0.179 |
| CHD (n, %) | 2,358 (69.98%) | 13,464 (69.45%) | 0.527 |
| Stroke (n, %) | 718 (19.80%) | 7,002 (37.5%) | <0.001 |
| AF (n, %) | 944 (26.03%) | 2,059 (10.62%) | <0.001 |
| PAD (n, %) | 156 (4.30%) | 1,084 (5.59%) | 0.002 |
| **Medication history** | | | |
| Insulin (n, %) | 373 (10.28%) | 1743 (8.99%) | 0.0013 |
| RAAS inhibitors (n, %) | 1740 (47.97%) | 8,230 (42.45%) | <0.001 |
| Antiplatelet drugs (n, %) | 2,362 (65.12%) | 15,349 (79.17%) | <0.001 |
| Statins (n, %) | 2659 (73.31%) | 16,443 (84.81%) | <0.001 |
| **Laboratory examination data** | | | |
| WBC (×10^9^/L) | 7.69 (5.59, 9.79) | 7.10 (5.51, 8.69) | <0.001 |
| Neutrophil (×10^9^/L) | 5.36 (4.41, 6.61) | 4.66 (3.71, 5.91) | <0.001 |
| Monocytes (×10^9^/L) | 0.50(0.40,0.61) | 0.45 (0.36, 0.58) | <0.001 |
| Lymphocyte (×10^9^/L) | 1.64(1.23,2.10) | 1.81(1.48,2.29) | <0.001 |
| Platelet (×10^9^/L) | 193 (157, 235) | 202 (168, 241) | <0.001 |
| HGB (g/L) | 128 (114, 141) | 137 (126, 149) | <0.001 |
| SCr (µmol/L) | 94.5 (81, 117) | 81.9 (69, 92) | <0.001 |
| BUN (mmol/L) | 6.4 (5.0, 8.4) | 5.4 (4.4, 6.6) | <0.001 |
| Serum uric acid (µmol/L) | 362 (296, 443) | 332 (275, 397) | <0.001 |
| eGFR (mL/min/1.73 m^2^) | 70.79 (54.41, 87.25) | 82.45 (68.37, 95.92) | <0.001 |
| Urine protein |  |  | <0.001 |
| 0-± | 2,849 (78.55%) | 17,682 (91.21%) |  |
| 1+-2+ | 636 (17.54%) | 1385 (7.14%) |  |
| 3+-4+ | 142 (3.92%) | 320 (1.65%) |  |
| Serum total protein (g/L) | 64 (60, 68) | 65 (61, 70) | <0.001 |
| Serum ALB (g/L) | 37.9 (35.2, 40.5) | 40.2 (37.7, 43.0) | <0.001 |
| Serum fibrinogen (g/L) | 3.26 (2.76, 3.83) | 3.05 (2.67, 3.52) | <0.001 |
| ALT (U/L) | 20 (13, 33) | 18 (13, 28) | <0.001 |
| AST (U/L) | 24 (19, 35) | 21 (17, 29) | 0.425 |
| Total bilirubin (µmol/L) | 14.5 (10.3, 19.3) | 13.3 (10.4, 17.2) | <0.001 |
| TC (mmol/L) | 3.93 (3.33, 4.76) | 4.04 (3.41, 4.78) | <0.001 |
| TG (mmol/L) | 1.36 (0.98, 2.01) | 1.42 (1.03, 1.96) | <0.001 |
| HDL-C (mmol/L) | 0.94 (0.80, 1.13) | 0.98 (0.84, 1.15) | <0.001 |
| LDL-C (mmol/L) | 2.35 (1.80 ,3.04) | 2.40 (1.85,3.05) | 0.025 |
| FBG (mmol/L) | 5.8 (4.9, 7.6) | 5.5 (4.9, 6.8) | <0.001 |
| HbA1c (%) | 6.10 (5.70,7.00) | 6.20 (5.80,7.10) | 0.009 |
| NT-proBNP (ng/mL) | 784.5 (222.0, 2,339.8) | 172.0 (66.4, 628.2) | <0.001 |
| **Imaging examination data** | | | |
| LVEF (%) | 55 (36, 61) | 69 (63, 72) | <0.001 |
| LVEDD (mm) | 51.0 (47.0, 55.0) | 48.0 (45.0, 51.2) | <0.001 |
| Outcome Event | 884(24.37%) | 1791(9.24) | <0.001 |

**Abbreviations:** BMI, body mass index; SBP, systolic blood pressure; DBP, diastolic blood pressure; DM, diabetes mellitus; MetS, metabolic syndrome; CHD, coronary heart disease; AF, atrial fibrillation; HF, heart failure; PAD, peripheral artery disease; RAAS, renin-angiotensin-aldosterone system; WBC, white bllod cell; HGB, hemoglobulin; SCr, serum creatinine; BUN, blood urea nitrogen; eGFR, estimated glomerular filtration rate; ALB, albumin; ALT, alanine aminotransferase; AST, aspartate aminotransferase; TC, total cholesterol; TG, triglyceride; HDL-C, high-density lipoprotein cholesterol; LDL-C, low-density lipoprotein cholesterol; FBG, fasting blood glucose; HbA1c, glycosylated hemoglobin; NT-proBNP, N-terminal pro-B-type brain natriuretic peptide; LVEF, left ventricular ejection fraction; LVEDD, left ventricular end-diastolic diameter.

**Table S3. Collinear analysis of potential risk factors.**

|  | VIF |
| --- | --- |
| Age | 2.169 |
| Gender | 1.222 |
| Smoke | 1.220 |
| BMI | 1.264 |
| SBP | 1.510 |
| DBP | 1.663 |
| Heart rate | 1.249 |
| Hypertension | 1.307 |
| DM | 1.601 |
| Hyperlipidemia | 1.289 |
| MS | 1.663 |
| CHD | 1.297 |
| Stroke | 1.106 |
| AF | 1.349 |
| HF | 1.276 |
| PAD | 1.019 |
| Insulin use | 1.166 |
| RAAS inhibitors use | 1.187 |
| Antiplatelet drugs use | 1.595 |
| Statin use | 1.679 |
| WBC | 11.536 |
| Neutrophil | 10.860 |
| Monocytes | 1.640 |
| Lymphocyte | 2.231 |
| Platelet | 2.080 |
| HGB | 1.790 |
| SCr | 5.226 |
| BUN | 2.735 |
| Serum uric acid | 1.671 |
| eGFR (mL/min/1.73 m^2^) | 5.176 |
| Urine protein | 1.398 |
| Serum total protein | 1.932 |
| Serum ALB | 1.577 |
| Serum fibrinogen | 1.523 |
| ALT | 1.205 |
| AST | 1.107 |
| TC | 4.803 |
| TG | 2.349 |
| HDL-C | 1.417 |
| LDL-C | 4.236 |
| FBG | 1.884 |
| HbA1c | 2.076 |
| NT-proBNP | 1.257 |
| LVEDD | 1.960 |
| LVEF | 1.940 |

**Abbreviations:** BMI, body mass index; SBP, systolic blood pressure; DBP, diastolic blood pressure; DM, diabetes mellitus; MetS, metabolic syndrome; CHD, coronary heart disease; AF, atrial fibrillation; HF, heart failure; PAD, peripheral artery disease; RAAS, renin-angiotensin-aldosterone system; WBC, white bllod cell; HGB, hemoglobulin; SCr, serum creatinine; BUN, blood urea nitrogen; eGFR, estimated glomerular filtration rate; ALB, albumin; ALT, alanine aminotransferase; AST, aspartate aminotransferase; TC, total cholesterol; TG, triglyceride; HDL-C, high-density lipoprotein cholesterol; LDL-C, low-density lipoprotein cholesterol; FBG, fasting blood glucose; HbA1c, glycosylated hemoglobin; NT-proBNP, N-terminal pro-B-type brain natriuretic peptide; LVEF, left ventricular ejection fraction; LVEDD, left ventricular end-diastolic diameter.

**Table S4. Potential risk factors identified by Cox regression analysis**

|  | | Univariate Cox regression | | Multivariate Cox regression | |
| --- | --- | --- | --- | --- | --- |
|  | | HR [95%CI] | *P* | HR [95%CI] | *P* |
| Age (per 1-year increase) | 1.026[1.022-1.030] | | ＜0.001 | 1.021[1.017-1.025] | ＜0.001 |
| Gender (male *vs.*female) | 1.023[0.952-1.099] | | 0.929 |  |  |
| Smoke | 1.012[0.967-1.061] | | 0.846 |  |  |
| BMI (per 1-Kg/ m^2^increase) | 0.979[0.970-0.987] | | ＜0.001 | 0.994[0.985-1.002] | 0.143 |
| SBP (per 1-mmHg increase) | 1.004[1.002-1.006] | | ＜0.001 | 1.005[1.003-1.008] | <0.001 |
| DBP (per 1-mmHg increase ) | 0.993[0.991-0.995] | | ＜0.001 | 0.998[0.995-1.001] | 0.192 |
| Heart rate (per 1-bpm increase ) | 1.014[1.011-1.018] | | ＜0.001 | 1.003[0.999-1.007] | 0.141 |
| Hypertension (yes *vs.*no) | 1.117[1.084-1.151] | | ＜0.001 | 1.210[1.176-1.253] | ＜0.001 |
| DM (yes *vs.*no) | 1.172[1.231-1.226] | | ＜0.001 | 1.230[1.175-1.296] | 0.001 |
| Hyperlipidemia (yes *vs.*no) | 1.003[1.002-1.004] | | ＜0.001 | 1.000[0.999-1.001] | 0.932 |
| MS (yes *vs.*no) | 1.083[0.963-1.197] | | 0.842 |  |  |
| CHD (yes *vs.*no) | 0.999[0.998-1.000] | | 0.050 |  |  |
| Stroke (yes *vs.*no) | 1.003[0.974-1.031] | | 0.732 |  |  |
| AF (yes *vs.*no) | 1.000[0.945-1.061] | | 0.960 |  |  |
| HF (yes *vs.*no) | 1.312[1.170-1.473] | | ＜0.001 | 1.265[1.205-1.338] | <0.001 |
| PAD (yes *vs.*no) | 0.973[0.917-1.031] | | 0.682 |  |  |
| Insulin use (yes *vs.*no) | 0.913[0.886-0.940] | | ＜0.001 | 0.984[0.957-1.011] | 0.254 |
| RAAS inhibitors use (yes *vs.*no) | 0.872[0.846-0.898] | | ＜0.001 | 0.812[0.790-0.848] | <0.001 |
| Antiplatelet drugs use (yes *vs.*no) | 0.783[0.764-0.802] | | ＜0.001 | 0.862[0.823-0.925] | <0.001 |
| Statin use (yes *vs.*no) | 0.902[0.876-0.928] | | ＜0.001 | 0.968[0.924-1.002] | 0.125 |
| WBC (per 1×10^9/L increase) | 1.028[1.023-1.032] | | ＜0.001 | 1.016[0.998-1.031] | 0.062 |
| Neutrophil (per 1×10^9/L increase) | 1.064[1.030-1.098] | | ＜0.001 | 1.032[0.987-1.078] | 0.164 |
| Monocytes (per 1×10^9/L increase) | 1.251[1.130-1.372] | | ＜0.001 | 1.058[0.972-1.144] | 0.172 |
| Lymphocyte (per 1×10^9/L increase) | 0.868[0.837-0.901] | | ＜0.001 | 0.962[0.921-1.002] | 0.073 |
| Platelet (per 1×10^9/L increase) | 1.000[0.999-1.002] | | 0.506 |  |  |
| HGB (per 1-g/L increase) | 0.979[0.975-0.983] | | ＜0.001 | 0.996[0.994-0.998] | ＜0.001 |
| SCr (per 1-umol/L increase) | 1.015[1.006-1.023] | | ＜0.001 | 1.018[0.999-1.037] | 0.061 |
| BUN (per 1-mmol/L increase) | 1.129[1.123-1.136] | | ＜0.001 | 1.048[1.038-1.061] | ＜0.001 |
| Serum uric acid (per 1-umol/L increase) | 1.010[1.002-1.019] | | ＜0.001 | 1.012[0.998-1.027] | 0.112 |
| eGFR (per 1-mL/min/1.73 m ^2^ increase) | 1.006[0.997-1.014] | | 0.162 |  |  |
| Urine protein | 1.784[1.691-1.878] | | ＜0.001 | 1.692[1.580-1.808] | ＜0.001 |
| Serum total protein (per 1-g/L increase) | 1.002[0.997-1.007] | | 0.433 |  |  |
| Serum ALB (per 1-g/L increase) | 0.904[0.899-0.910] | | ＜0.001 | 0.965[0.956-0.976] | ＜0.001 |
| Serum  Fibrinogen (per 1-g/L increase) | 1.219[1.075-1.366] | | ＜0.001 | 1.159[1.073-1.249] | ＜0.001 |
| ALT (per 1-U/L increase) | 1.004[0.997-1.012] | | 0.291 |  |  |
| AST (per 1-U/L increase) | 1.001[0.995-1.008] | | 0.762 |  |  |
| TC (per 1-mmol/L increase) | 1.006[0.995-1.018] | | 0.305 |  |  |
| TG (per 1-mmol/L increase) | 1.042[1.024-1.061] | | ＜0.001 | 1.086[1.062-1.112] | ＜0.001 |
| HDL-C (per 1-mmol/L increase) | 1.065[1.049-1.082] | | ＜0.001 | 1.006[0.981-1.032] | 0.642 |
| LDL-C (per 1-mmol/L increase) | 1.052[1.038-1.067] | | ＜0.001 | 1.011[0.989-1.035] | 0.351 |
| FBG (per 1-mmol/L increase) | 1.006[0.989-1.028] | | 0.544 |  |  |
| HbA1c (per 1% increase) | 1.087[1.061-1.114] | | ＜0.001 | 1.059[1.037-1.082] | ＜0.001 |
| NT-proBNP (per 1-pg/mL increase) | 1.064[0.948-1.193] | | 0.287 |  |  |
| LVEDD (per 1-mm increase) | 1.027[1.024-1.031] | | ＜0.001 | 1.001[0.995-1.008] | 0.763 |
| LVEF (per 1% increase) | 0.972[0.967-0.978] | | ＜0.001 | 0.969[0.962-0.977] | ＜0.001 |

Due to collinearity analysis indicating variance inflation factor (VIF) > 5, neutrophils and eGFR were excluded from the multivariate Cox regression analysis. Multivariate Cox’s regression model included all the significant variables (p<0.1) from the univariate analysis.

**Abbreviations**: BMI, body mass index; SBP, systolic blood pressure; DBP, diastolic blood pressure; DM, diabetes mellitus; MetS, metabolic syndrome; CHD, coronary heart disease; AF, atrial fibrillation; HF, heart failure; PAD, peripheral artery disease; RAAS, renin-angiotensin-aldosterone system; WBC, white bllod cell; HGB, hemoglobulin; Scr, serum creatinine; BUN, blood urea nitrogen; eGFR, estimated glomerular filtration rate; ALB, albumin; ALT, alanine aminotransferase; AST, aspartate aminotransferase; TC, total cholesterol; TG, triglyceride; HDL-C, high-density lipoprotein cholesterol; LDL-C, low-density lipoprotein cholesterol; FBG, fasting blood glucose; HbA1c, glycosylated hemoglobin; NT-proBNP, N-terminal pro-B-type brain natriuretic peptide; LVEF, left ventricular ejection fraction; LVEDD, left ventricular end-diastolic diameter

**Table S5.Comparison of predictor effects between Cox model and Fine-Gray competing risk model for predicting kidney function progression**

| **Variables** | **Cox Model** | | **Fine-Gray Model** | |
| --- | --- | --- | --- | --- |
|  | **HR (95% CI)** | ***P*** | **SHR (95% CI)** | ***P*** |
| Age (per 1-year increase) | 1.021[1.017-1.025] | ＜0.001 | 1.028[1.022-1.032] | ＜0.001 |
| SBP (per 1-mmHg increase) | 1.005[1.003-1.008] | <0.001 | 1.005[1.003-1.008] | <0.001 |
| Hypertension (yes *vs.*no) | 1.210[1.176-1.253] | <0.001 | 1.224[1.190-1.257] | <0.001 |
| DM (yes *vs.*no) | 1.230[1.175-1.296] | 0.001 | 1.241[1.186-1.324] | 0.001 |
| HF (yes *vs.*no) | 1.265[1.205-1.338] | <0.001 | 1.373[1.314-1.448] | <0.001 |
| RAAS inhibitors use (yes *vs.*no) | 0.812[0.790-0.848] | <0.001 | 0.804[0.782-0.837] | <0.001 |
| Antiplatelet drugs use (yes *vs.*no) | 0.862[0.823-0.915] | <0.001 | 0.879[0.837-0.928] | <0.001 |
| HGB (per 1-g/L increase) | 0.996[0.994-0.998] | <0.001 | 0.998[0.996-1.000] | 0.514 |
| BUN (per 1-mmol/L increase) | 1.048[1.038-1.061] | <0.001 | 1.036[1.025-1.050] | <0.001 |
| Urine protein | 1.692[1.580-1.808] | <0.001 | 1.721[1.609-1.842] | <0.001 |
| Serum ALB (per 1-g/L increase) | 0.965[0.956-0.976] | <0.001 | 0.951[0.942-0.963] | <0.001 |
| Fibrinogen (per 1-g/L increase) | 1.159[1.073-1.249] | <0.001 | 1.276[1.193-1.367] | <0.001 |
| TG (per 1-mmol/L increase) | 1.086[1.062-1.112] | <0.001 | 1.074[1.050-1.101] | <0.001 |
| HbA1c (per 1% increase) | 1.059[1.037-1.082] | <0.001 | 1.063[1.042-1.087] | <0.001 |
| LVEF (per 1% increase) | 0.969[0.962-0.977] | <0.001 | 0.957[0.949-0.966] | <0.001 |

**Abbreviations:** SBP, systolic blood pressure; DM, diabetes mellitus; HF, heart failure; RAAS, renin-angiotensin-aldosterone system; HGB, hemoglobulin; BUN, ALB, albumin; TG, triglyceride; HbA1c, LVEF, left ventricular ejection fraction.

**Table S6. The results of LASSO-Cox regression**

| **Variables** | **HR** | **95%CI** | ***P*-value** |
| --- | --- | --- | --- |
| Age (incremented by 1 year) | 1.027 | 1.022-1.032 | <0.001 |
| DM (yes *vs.*no) | 1.214 | 1.084-1.358 | 0.007 |
| Hypertension (yes *vs.*no) | 1.145 | 1.015-1.290 | 0.027 |
| AF (yes *vs.*no) | 1.248 | 1.099-1.404 | 0.004 |
| HF (yes *vs.*no) | 1.298 | 1.156-1.460 | <0.001 |
| Usage of antiplatelet drugs (yes *vs.*no) | 0.875 | 0.788-0.971 | 0.012 |
| SBP (incremented by 1 mmHg) | 1.006 | 1.004-1.009 | <0.001 |
| HGB (incremented by 1 g/L) | 0.982 | 0.979-0.985 | <0.001 |
| Fibrinogen (incremented by 1 g/L) | 1.081 | 1.027-1.137 | 0.001 |
| TG (incremented by 1 mmol/L) | 1.089 | 1.058-1.121 | <0.001 |
| HbA1c (incremented by 1%) | 1.096 | 1.061-1.132 | <0.001 |
| Serum ALB (incremented by 1 g/L) | 0.967 | 0.956-0.978 | <0.001 |
| Serum uric acid (incremented by 1 µmol/L) | 1.001 | 1.001-1.002 | <0.001 |
| Urine protein | 1.542 | 1.409-1.684 | <0.001 |
| LVEF (incremented by 1%) | 0.968 | 0.964-0.971 | <0.001 |

**Abbreviations:** DM, Diabetes Mellitus; AF, atrial fibrillation; HF, heart failure; SBP, systolic blood pressure; HGB, hemoglobulin; TG, Triglyceride;HbA1c, glycosylated hemoglobin;ALB, Albumin; LVEF, left ventricular ejection fraction.

**TableS7. The number of at-risk individuals and censored individuals at each time point in the time-dependent ROC curve of the validation cohort**

| **Time Point** | **Number at Risk** | **Censored** |
| --- | --- | --- |
| 12 months | 7410 | 16 |
| 24 months | 7091 | 37 |
| 36 months | 6681 | 65 |

**Abbreviations:** ROC,Receiver Operating Characteristic

**Table S8. Performance Comparison of RSF Model and KFRE Model**

| **Variables** | **KFRE Model** | **RSF model** |  |
| --- | --- | --- | --- |
| C-index (95% CI) | 0.852 (0.846-0.859) | 0.890 (0.883-0.896) | *P*<0.001 |
| For predicting 2-year risk |  |  |  |
| AUROC (95% CI) | 0.890 (0.882-0.899) | 0.921 (0.912-0.929) | *P*<0.001 |
| NRI | Reference | 0.22 (0.08-0.34) | *P*<0.05 |
| NRI:events | Reference | 0.15 (0.06-0.23) | *P*<0.05 |
| NRI:nonevents | Reference | 0.07 (0.03-0.12) | *P*<0.05 |
| IDI | Reference | 0.08 (0.05-0.11) | *P*<0.001 |

**Abbreviations:** CI, confidence interval; C-index, concordance index; IDI, integrated discrimination improvement; KFRE, Kidney Failure Risk Equation; NRI, net reclassification improvement; RSF, Random Survival Forest.

**The web address** for the web-based risk calculator based on the RSF model: https://fast.statsape.com/tool/detail?id=1
